# Dancing with the Dust Devil: Examining the Lung Mycobiome of Sonoran Desert Wild Mammals and the Effect of *Coccidioides* Presence

**DOI:** 10.3390/pathogens14080807

**Published:** 2025-08-14

**Authors:** Ana Fabio-Braga, Jaida Salois, Mitchell L. Bryant, Daniel R. Kollath, Bridget Barker

**Affiliations:** Pathogen and Microbiome Institute, Northern Arizona University, Flagstaff, AZ 86011, USA; ana.braga@nau.edu (A.F.-B.); jys27@nau.edu (J.S.); mitchellbryant@live.com (M.L.B.); daniel.kollath@nau.edu (D.R.K.)

**Keywords:** *Coccidioides*, coccidioidomycosis, life cycle, endozoan, animal reservoir, disease ecology, fungal pathogens, mycobiome, Onygenales, microbiome

## Abstract

Microbiome studies report a decrease in diversity associated with active infections. Under the endozoan hypothesis, *Coccidioides* can inhabit a host without causing disease. In this study, we describe and compare the lung mycobiome of *Coccidioides*-positive and -negative samples obtained from wildlife. If *Coccidioides* is not causing infection, we predict there will be no differences in the mycobiome between positive and negative samples. Lung samples were obtained from mammals previously trapped in Tucson, Arizona, USA (*n* = 26), and Mesa, Arizona, USA (*n* = 14). Samples were screened for *Coccidioides* with CocciDx, and mycobiome was characterized through Illumina-based amplicon sequencing of the internal transcribed spacer 2 (ITS2). We compared alpha and beta diversity of the mycobiome to assess the effects of *Coccidioides’* presence and host taxonomy. A greater number of reads were captured from Tucson samples (114,706.4 ± 57,945.8) than from Mesa (384.9 ± 953.5); however, Mesa (16.8 ± 8.8) and Tucson (12 ± 7.8) had a similar number of fungal genera per sample. CocciDx detected *Coccidioides* in more samples than the ITS2 amplicon sequencing. All samples from Mesa and five from Tucson tested positive for *Coccidioides*. Therefore, Mesa samples were excluded from statistical analysis. No difference in alpha and beta diversity was associated with *Coccidioides* presence, which is consistent with the endozoan hypothesis. Host taxonomy had a significant effect on beta diversity. This effect is likely driven by host behavioral and physiological differences.

## 1. Introduction

While there is an emerging interest in lung mycobiome of wild animals [1,2,3,4], we still have a limited understanding of the complex host-mycobiome dynamics [3,4]. Additionally, our understanding of microbiome dynamics is primarily based on other tissue systems (e.g., gut, vagina, mouth, etc.). Those studies suggest a decrease in the diversity of microbial communities associated with active infection. However, the role of the microbiome in disease progression is not well characterized [5,6,7,8,9].

The lungs are a very dynamic environment. Organisms enter the airways through inhalation or contact with the gut and are eliminated through coughing, mucociliary transport, innate and adaptive immune system responses [10]. Therefore, microorganisms found in the lungs could be transient or part of the established microbial community, leading to complications in analyzing data and drawing conclusions [3,11,12]. Moreover, many of these studies have few healthy controls (or none) due to the invasive nature of collection of bronchoalveolar lavage fluid or biopsy specimens. Finally, in human and laboratory animal studies, differences in microbiome composition are associated with multiple factors such as age, mode of birth, health, diet, and other lifestyle factors [13,14,15,16,17].

Initially, studies described the diversity of fungi in mammalian lungs and their role as pathogens through culturing [18,19,20,21]. However, culture-based approaches have been shown to be ineffective in capturing total microbial diversity in the lung. In one study, 60% of the species detected with pyrosequencing were not recovered through culturing from clinical samples in patients with cystic fibrosis [22]. Community sequencing is a valuable tool to characterize microbial communities that more recently have been used to look at the mycobiome associated with wildlife [3,23,24,25,26,27,28,29,30,31].

Coccidioidomycosis is an environmentally acquired fungal disease caused by *Coccidioides posadasii* and *Coccidioides immitis* [32]. Most cases of the disease are asymptomatic and are often misdiagnosed [33,34,35,36]. Yet, 17,612 cases of coccidioidomycosis were reported in the United States in 2022, the majority in Arizona and California [37]. A study using data from 2019 estimated that the lifetime cost of coccidioidomycosis would be 736 million USD for the 10,539 cases diagnosed in Arizona in that year [38]. *Coccidioides* acts as a pathogen in humans and other mammals, such as dogs, horses, and even sea lions [39]. However, evidence also suggests that the fungus may behave as an “endozoan”, residing in the tissues of small mammals without causing coccidioidomycosis, similar to the relationship between endophytes and their plant hosts [40,41]. If this is the case, small mammals may serve as important reservoirs for *Coccidioides* in the environment, but additional evidence is required to resolve the role of small mammals on the *Coccidioides* life cycle [40,42,43,44,45].

Lung microbiome studies could provide valuable insights into the behavior of *Coccidioides* in small mammals. If *Coccidioides* behaves as an endozoan, there should be no significant difference in the microbiome of *Coccidioides*-positive and -negative lungs. To date, only one study has examined the impact of *Coccidioides* on the lung microbiome. This study found no significant differences associated with *Coccidioides* presence in the lungs of small mammals obtained from a museum collection [3]. Given the limited research on wildlife mycobiome and the effect of *Coccidioides* in the microbial communities, the overarching goal of this study is to characterize the mycobiome of small-mammal lung samples from *Coccidioides*-endemic areas and identify differences associated with the presence of *Coccidioides*.

## 2. Methods

### 2.1. Samples

All lung samples were obtained from specimens donated to this study postmortem (Scientific Collection License #SP650076). Animals were identified based on morphological characteristics. The Arizona Game and Fish Department donated lungs of *Dipodomys* (kangaroo rat, *n* = 1), *Chaetodipus* (pocket mouse, *n* = 12), *Onychomys* (grasshopper mouse, *n* = 1), *Ammospermophilus* (antelope ground squirrel, *n* = 1), *Xerospermophilus* (round-tailed ground squirrel, *n* = 1), *Sylvilagus* (desert cottontail, *n* = 6), *Lepus* (antelope jackrabbit, *n* = 1), and *Lepus* (black-tailed jackrabbit, *n* = 3). Animals were euthanized on site and whole carcasses or lung tissue samples were stored temporarily in ice and later at −20 °C. Traps were set within an area of approximately 0.54 hectares located southeast of Tucson in a Lower Sonoran Desert vegetation area. Vegetation was sparse with mainly *Prosopis velutina* (mesquite tree), *Cercidium* spp. (palo verde tree) and *Larrea tridentata* (creosote bush).

*Peromyscus* (cactus mouse, *n* = 14), trapped for pest-control inside a building (approx. 184.3 m^2^), were donated by a facility north of Mesa in the Phoenix metropolitan area. Traps were checked at least twice a day, and carcasses were individually placed in a plastic bag and froze at −20 °C. The area where these animals were obtained is a run-off zone of the Salt River with abundant wildlife (e.g., wild horses, horned owls, coyotes, and diverse communities of snakes and burrowing mammals). Vegetation in the area is sparse and includes *Larrea tridentata* (creosote bush), *Encelia farinose* (brittle brush), and *Prosopis velutina* (mesquite tree). Invasive grass like *Cenchrus ciliaris* (buffelgrass) occupies spaces in the spring and summer.

*Coccidioides* was known to be present in the Mesa site, as an ongoing study screened soil samples monthly from 2022 to 2025 [46]. However, for the Tucson site it was necessary to determine if *Coccidioides* was present in the soil. Soil samples were collected from animal burrow entrances using a garden trowel or long handled kitchen spoon and sterilized with 10% bleach between soil samples. Soil was put directly into sterile 50-milliliter specimen cups. Soil was stored at room temperature until DNA extraction was performed. In total, 48 soil samples were collected from four unique burrow systems (12 replicates per burrow system).

### 2.2. DNA Extraction

Animal carcasses or tissues were kept in a −20 °C freezer until transferred to Northern Arizona University. Carcasses were then necropsied in the laboratory. Following necropsy, tissues were kept in a −80 °C freezer until DNA extractions were completed. Tissues were placed in bead tubes (BeadTube 1.5 mm, Benchmark Scientific, Sayreville, NJ, USA) and homogenized at 5 m/s for one minute. For Mesa site samples, DNA extractions were conducted using a phenol-chloroform extraction protocol. For Tucson site samples, DNA was extracted with Qiagen Blood and Tissue Kit (Qiagen, Valencia, CA, USA) following the manufacturer’s protocol.

DNA from soil samples was extracted using the DNeasy PowerSoil Pro kit (QIAGEN, Valencia, CA). The manufacturer’s protocol was followed with the addition of a 10 min heat step at 65 °C before homogenization [47]. For each soil sample, DNA was extracted from two different 250 mg sub-samples. All DNA was stored in a −20 °C freezer.

### 2.3. Molecular Detection of Coccidioides

The CocciDx assay was used to determine whether lung and soil samples were positive or negative for *Coccidioides.* This assay is a real-time qPCR targeting a 106 bp sequence present in multiple copies within a transposable element in the genome of both *Coccidioides* species and only present in the *Coccidioides* genus [48,49]. CocciDx was performed on the Applied Biosystems QuantStudio 12K Flex Real-Time PCR System (Thermo Fisher Scientific, Waltham, MA, USA). Each reaction contained 2× TaqMan Environmental Master Mix 2.0 (Thermo Fisher Scientific, Waltham, MA, USA), 1× CocciDX at 100μM concentration of Cocci Assay oligo/probe mix and 2 μL of DNA extracted from lung sample. Each reaction had a total volume of 20 uL. PCR cycling conditions were two minutes at 50 °C for the activation step, 10 min at 95 °C for the denaturation step, 45 cycles of 15 s at 95 °C and then one minute at 60 °C. Samples are run in triplicate with molecular grade water as a negative control and Silveira strain DNA as positive control. Samples with CT values lower than 40 were considered positive.

### 2.4. Amplicon Sequencing

To determine the composition of fungal communities, each DNA extract went through an Illumina-based amplicon sequencing, targeting the ITS2 region. We used 5.8S-Fun (ACCCAACTGAATGGAGCAACTTTYRRCAAYGGATCWCT) and ITS4-Fun (ACGCACTTGACTTGTCTTCAGCCTCCGCTTATTGATATGCTTAART) as primers and added universal tail sequences [50].

The amplification reaction of the fungal ITS2 region contained 1X Phusion Green Hot Start II High-Fidelity PCR Master Mix (Thermo Fisher Scientific, Waltham, MA, USA), 0.5 μM each primer, 2 μL of DNA and molecular grade water to a final volume of 50 μL. A universal tail was added to the forward and reverse primer to facilitate amplicon sequencing [51]. Cycling parameters were initial denaturation at 98 °C for 1 min followed by 30 cycles of 98 °C denaturation for 10 s, 57 °C to 59 °C annealing for 30 s, and 72 °C extension for 20 s. A final extension consisted of 10 min at 72 °C. The success of the amplifications was assessed by running the PCR products on a 2% agarose gel. Amplicons for each sample were cleaned using 1X Agencourt AMPure XP beads (Beckman Coulter, Brea, CA, USA) as recommended by the manufacturer. Unique indices and sequencing adaptors were added during a second PCR reaction that uses the universal tails attached during the initial PCR. This reaction consisted of 12.5 μL of Kapa HiFi HotStart Ready Mix (Kapa Biosystems, Wilmington, MA, USA), 400 nm of the unique indexing primers, and 2, 4, or 8 μL of the bead-cleaned amplicons from the initial PCR. Thermocycling conditions were 2 min at 98 °C for initial denaturation, 6 cycles of: 30 s at 98 °C denaturation, 20 s at 60 °C annealing, 30 s at 72 °C extension, and a final extension at 72 °C for 5 min. Indexed libraries were pooled in equal amounts (240 ng) using the Qubit fluorometer (Thermo Fisher Scientific, Waltham, MA, USA). The combined pool was cleaned with the QIAquick PCR Purification Kit (Qiagen, Valencia, CA, USA) per manufacturer’s recommendations. The samples were sequenced on an Illumina MiSeq instrument using a v2 500 cycle kit (Illumina, San Diego, CA, USA).

### 2.5. Microbiome Data Processing

Primer sequences were trimmed from raw sequences using Cutadapt and paired end reads merged prior to data analysis [52]. Sequences were trimmed uniformly to 300 nucleotides. ITS2 samples were demultiplexed in Quantitative Insights Into Microbial Ecology 2 [53] requiring 95% of each read to have a minimum *q*-score of 20, and allowing no exceptions (-*q* 19 -*r* 0 -*p* 0.95). Forward reads were trimmed of primer sequence, followed by quality control filtering by DADA2 [54]. Then, demultiplexed, trimmed sequences were screened for chimeras using the VSEARCH program [55] and screened for fungal ITS2 sequences with ITSx [56]. Operational taxonomic units (OTUs) were assigned taxonomy using BLAST+ 2.15.0 [57] against the UNITE reference database [58]. Sequences were submitted to NCBI Genbank under the Bioproject PRJNA1235246.

### 2.6. Animal-Associated Fungal Genera

To distinguish between transient and established fungal genera in the lung mycobiome, we assigned the fungal genera into categories based on ecological traits. If a genus was not reported to be associated with vertebrate animals, we assume this genus is transiently present in the lung samples. Whereas, if a genus has been found to be associated with vertebrate animals, we assume this genus could be established in the mycobiome. To obtain information on the ecology of each genus, we used the FUNGuild database [59] and expanded upon this foundation by incorporating the latest scientific literature. Then, information was cross-referenced with the FUNGuild database and this amended database was used to assign the mycobiome genera into one of the following groups: animal associated, plant associated, dung associated, soil associated, other, unknown and NA. Fungal genera were only assigned into one group. The goal was to identify genera previously reported to be associated with vertebrate animals, thus if a fungal genus could be assigned to multiple functional categories (e.g., animal and plant associated), the animal associated category prevailed. Genus was assigned as unknown if no ecological data was available for that genus. OTUs that could not be identified to the genus level were described as NA.

### 2.7. Statistical Analysis

Given the difference in extraction methods and location where samples were obtained, the data from Mesa and Tucson sites are presented separately. Comparisons between *Coccidioides*-positive and -negative samples were only performed using the Tucson samples. Diversity analyses were performed at genus and order levels; however, not all OTUs had taxonomic assignments at genus or order. In these cases, we maintained the most specific taxonomic assignment. Abundance plots display the ten most prevalent fungal taxa for clarity, but statistical analysis were performed with the whole dataset.

To understand whether diversity patterns can be related to *Coccidioides* presence, the Bray–Curtis measure of dissimilarity (Beta-diversity) and the Shannon diversity index (Alpha diversity) were calculated for the Tucson dataset. For alpha diversity comparisons, after checking for normality, we either performed a two-tailed t-test or a Wilcoxon rank-sum test (nonparametric). For beta diversity comparisons, we assessed the homogeneity of variances in the data distribution. Then we compared the beta diversities with a permutational multivariate analysis of variance for distance matrices (PERMANOVA). All statistical analyses were conducted in R 4.3.3 with the packages Microbiome 1.26.0 [60], Phyloseq 1.48.0 [61], Ggplot2 3.5.1 [62], Vegan 2.6-6.1 [63], Tidyverse 2.0.0 [64], Dplyr 1.1.4 [65], and the FunGuild database [59].

## 3. Results

### 3.1. Coccidioides Detection

All lungs from *Peromyscus* (*n* = 14), obtained in the Mesa site tested positive for *Coccidioides*. For the samples obtained in the Tucson site, one *Ammospermophilus*, two *Chaetodipus*, one *Sylvilagus* and one *Onychomys* tested positive for *Coccidioides* (Table 1). Amplicon sequencing detected *Coccidioides* in six samples, but it was not as sensitive as the CocciDx assay which detected *Coccidioides* in 19 samples (Table 1). From the amplicon sequencing analysis *Coccidioides* was identified in two *Chaetodipus* (PM_4 and PM_5) and in four *Peromyscus* (33L, 34L, 35L, 38L). In the soil samples from the Tucson site (*n* = 48), 10.4% of the soils tested positive for *Coccidioides.* Soils that tested positive were obtained from two different burrow systems, two samples from burrow system B1 and three from burrow system B2 (Table S1).

### 3.2. Fungal Microbiome

**Figure 1:**
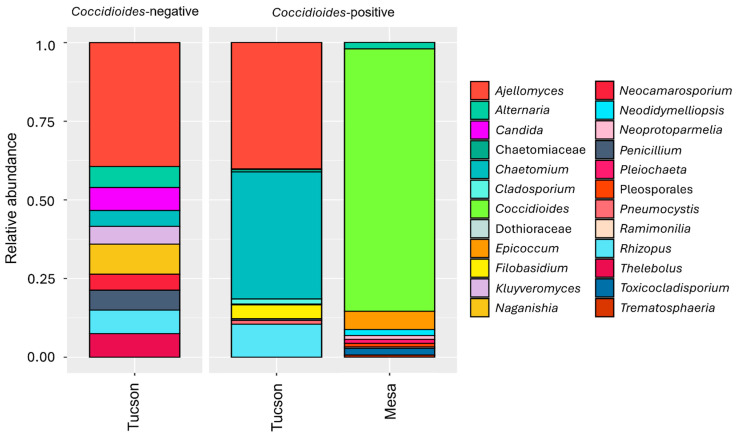
The ten most prevalent genera in the lung mycobiome are plotted for each site when samples are positive or negative for *Coccidioides*. In the Tucson site (*n* = 26), the ten most prevalent fungal genus are *Ajellomyces* (34.8%), *Chaetomium* (10.9%), *Rhizopus* (7.1%), *Naganishia* (6.7%), *Thelebolus* (5.2%), *Candida* (5.1%), *Alternaria* (4.7%), *Penicillium* (4.5%), *Kluyveromyces* (4.0%), *Neocamarosporium* (3.5%). In the Mesa site (*n* = 14), the most prevalent fungal genus are *Coccidioides* (78.7%), *Epicoccum* (5.5%), *Toxicocladosporium* (2.0%), *Alternaria* (1.9%), *Neodidymelliopsis* (1.8%), *Pleiochaeta* (1.2%), *Neoprotoparmelia* (1.1%), Pleosporales (1.1%), *Trematosphaeria* (0.6%) and *Ramimonilia* (0.4%).

**Figure 2:**
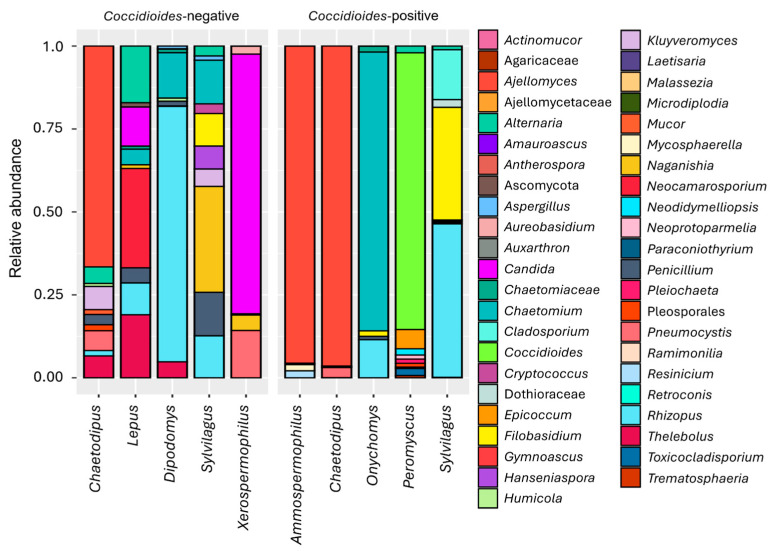
The ten most prevalent fungal genera in lung mycobiome plotted for each host genus when they are negative or positive for *Coccidioides*. In ***Dipodomys*** (*n* = 1) the most abundant genera were *Rhizopus* (76.9%), *Chaetomium* (13.7%), *Thelebolus* (4.8%), *Penicillium* (1.5%), Chaetomiaceae (1.1%); in ***Onychomys*** (*n* = 1), *Chaetomium* (84.1%), *Rhizopus* (11.5%), Chaetomiaceae (1.8%), *Filobasidium* (1.6%), *Penicillium* (1.0%); in ***Ammospermophilus*** (*n* = 1), *Ajellomyces* (95.6%), *Resinicium* (2.1%), *Mycosphaerella* (1.9%), Ascomycota (0.4%); in ***Xerospermophilus*** (*n* = 1), *Candida* (78.3%), *Pneumocystis* (14.3%), *Naganishia* (4.6%), *Aureobasidium* (2.4%), *Chaetomium* (0.4%); in ***Chaetodipus*** (*n* = 12), *Ajellomyces* (68.1%), *Kluyveromyces* (5.8%), *Thelebolus* (5.4%), *Pneumocystis* (5.3%), *Alternaria* (4.1%); in ***Sylvilagus*** (*n* = 6), *Naganishia* (28.4%), *Rhizopus* (15.3%), *Chaetomium* (11.7%), *Penicillium* (11.7%), *Filobasidium* (11.7%); in ***Lepus*** (*n* = 4), *Neocamarosporium* (28.9%), *Thelebolus* (18.4%), *Alternaria* (16.5%), *Candida* (11.4%), *Rhizopus* (9.3%). In ***Peromyscus*** (*n* = 14; Mesa site), the most abundant genera are *Coccidioides* (78.7%), *Epicoccum* (5.5%), *Toxicocladosporium* (2.0%), *Alternaria* (1.9%), *Neodidymelliopsis* (1.8%), *Pleiochaeta* (1.2%), *Neoprotoparmelia* (1.1%), Pleosporales (1.1%), *Trematosphaeria* (0.6%) and *Ramimonilia* (0.4%).

**Figure 3:**
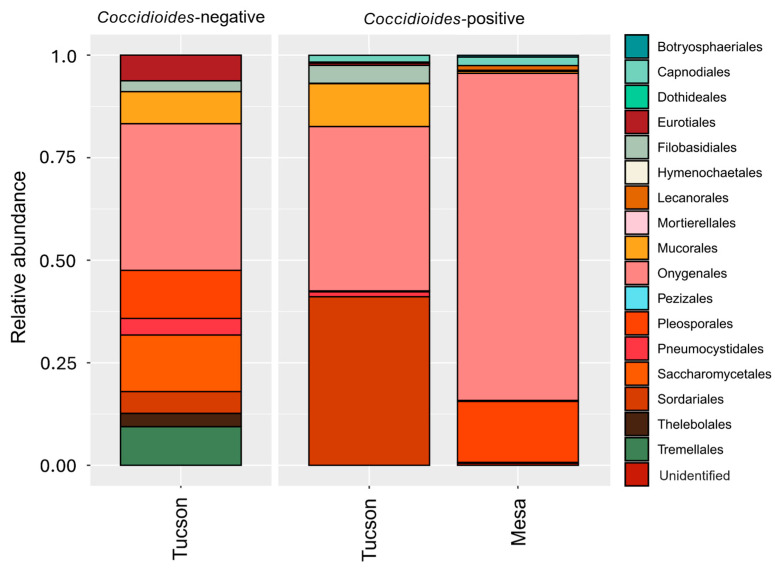
The ten most prevalent orders in the lung mycobiome are plotted for each site when samples are positive or negative for *Coccidioides*. In the Tucson site (*n* = 26), the most prevalent fungal orders are Onygenales (36.1%), Sordariales (12.0%), Saccharomycetales (11%), Pleosporales (9.4%), Mucorales (8.2%), Tremellales (7.5%), Eurotiales (5.1%), Pneumocystidales (3.5%), Filobasidiales (2.9%), and Thelebolales (2.6%). In the Mesa site (*n* = 14), the most prevalent fungal orders are Onygenales (78.8%), Pleosporales (14.7%), Capnodiales (2.1%), Lecanorales (1.1%), Unidentified (0.5%), Mucorales (0.5%), Botryosphaeriales (0.4%), Mortierellales (0.3%), Pezizales (0.2%) and Sordariales (0.2%) and Spizellomycetales (0.2%).

In the Mesa site, we identified 5389 amplicon sequences across the 14 samples from different *Peromyscus* individuals. We found an average of 384.9 ± 953.5 amplicon reads per sample with 16.8 ± 8.8 genus identified. The ten most prevalent genera accounted for 94.3% of the total reads (Figure 1 and Figure 2), while the ten most prevalent orders, accounted for 98.9% of the total reads (Figure 3). Given the limited sampling depth in the Mesa site samples, we only report fungal taxa and their relative abundances. In the Tucson site, 2,982,366 amplicon sequences were identified across 26 samples of different individuals belonging to distinct species. On average there were 114,706.4 ± 57,945.8 amplicon reads per sample with 12 ± 7.8 genus identified. The ten most prevalent genera accounted for 86.5% of the total reads (Figure 1 and Figure 2), while the ten most prevalent orders identified accounted for 98.4% of the total reads (Figure 3).

### 3.3. Comparisons of Alpha and Beta Diversity

**Figure 4:**
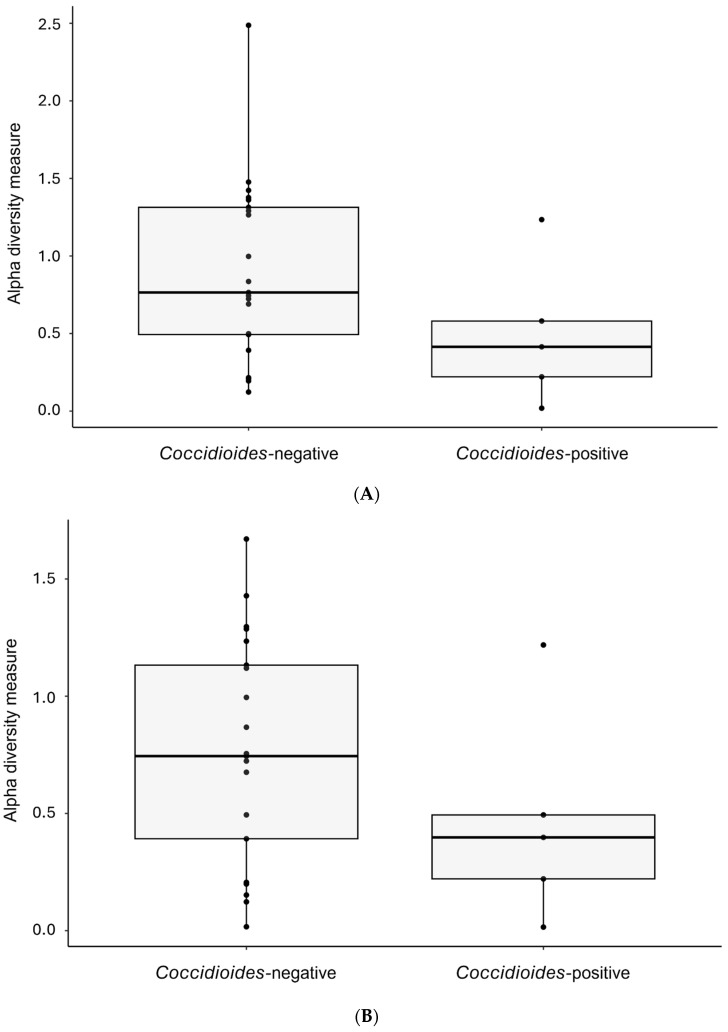
Alpha diversity comparisons calculated with the Shannon diversity index (Tucson samples only). (**A**) Alpha diversity plot at genus level. There are no differences in alpha diversity between *Coccidioides*-positive and -negative samples (*p* = 0.138). (**B**) Alpha diversity plot at order level. There are no differences in alpha diversity between *Coccidioides*-positive and -negative samples (*p* = 0.209).

**Figure 5:**
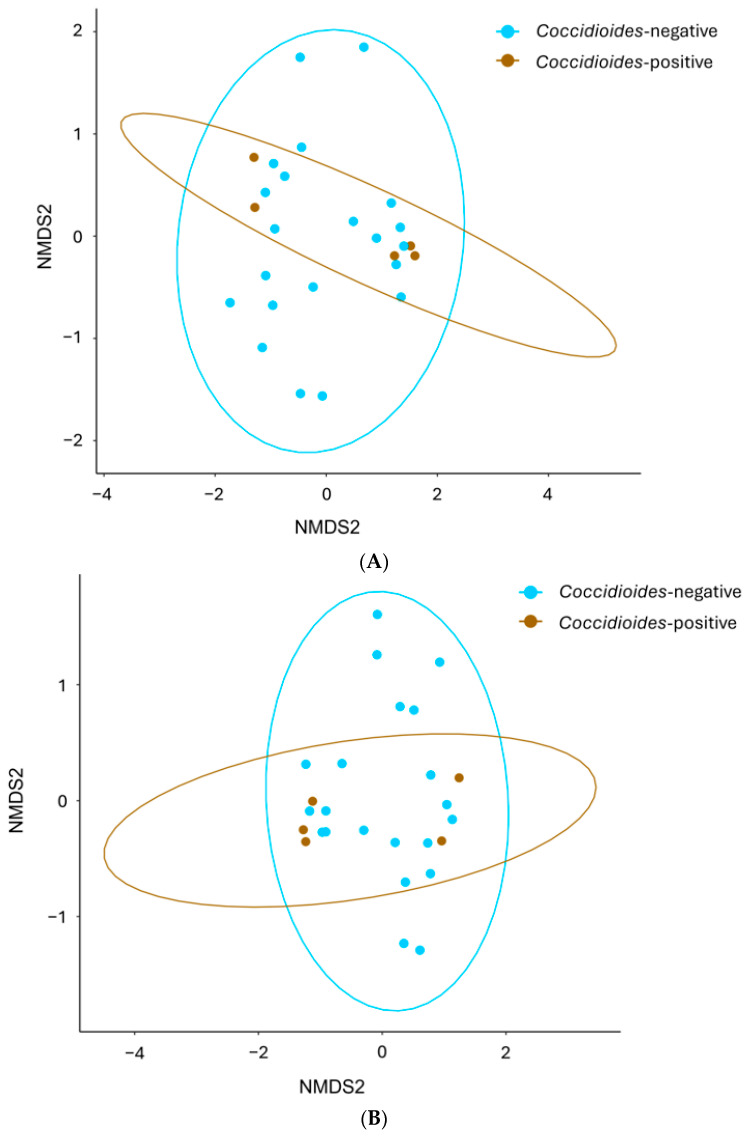
Beta diversity comparisons calculated with the Bray–Curtis measure of dissimilarity (Tucson samples only). (**A**) Beta diversity plot at genus level. There are no differences in beta diversity between *Coccidioides*-positive and -negative samples (*p* = 0.487). (**B**) Beta diversity plot at order level. There are no differences in beta diversity between *Coccidioides*-positive and -negative samples (*p* = 0.333).

**Figure 6:**
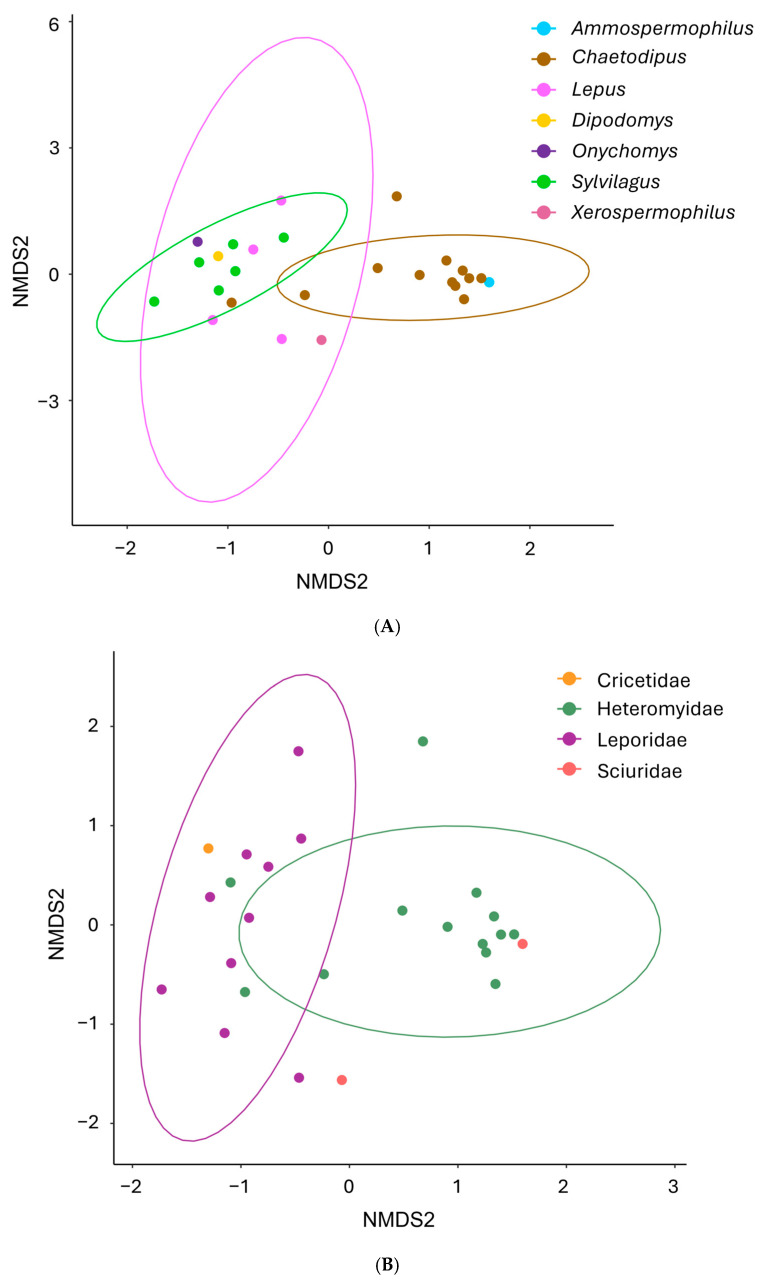
Beta diversity comparisons at genus level calculated with the Bray–Curtis measure of dissimilarity (Tucson samples only). (**A**) Host genus has a significant effect on lung mycobiome beta diversity (*p* = 0.001). (**B**) Host family has a significant effect on lung mycobiome beta diversity (*p* = 0.001).

There were no significant differences in alpha diversity assessed by the Shannon diversity index between *Coccidioides*-positive and -negative samples at the genus (*p* = 0.138) or at the order level (*p* = 0.209, Figure 4). No differences in beta diversity were found between *Coccidioides*-positive and -negative samples when OTUs are grouped at the genus level (*p* = 0.487) or at the order level (*p* = 0.333, Figure 5). Host family showed a significant effect on beta diversity both when OTUs are grouped at the genus level (*p* = 0.001) and at the order level (*p* = 0.003; Figure 6). Similarly, the host genus has a significant effect on beta diversity when OTUs are grouped at genus (*p* = 0.001) and order levels (*p* = 0.002; Figure 6).

### 3.4. Animal-Associated Fungal Community

**Figure 7:**
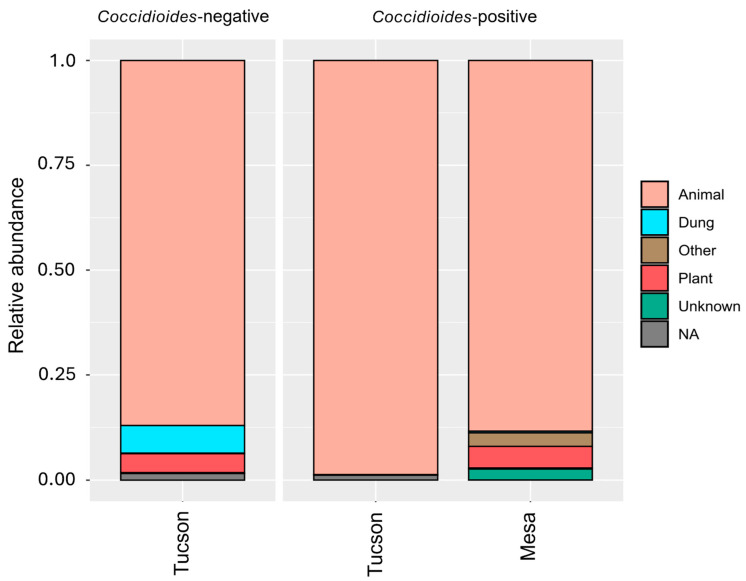
Assignment of fungal genera into functional categories: animal associated, plant associated, dung associated, other, unknown (no information available), or NA (OTU not identified to genus). Animal associated genera represent 89.2% of the original dataset for the Tucson samples and 88.3% for the Mesa samples.

Most of the genera found in the samples were previously reported to be associated with vertebrates (Figure 7). The animal-associated dataset represents 89.2% of the original reads in the Tucson samples (2,659,415 reads) and 88.3% in the Mesa samples (4761 reads). For Tucson, it resulted in an average of 102,285.2 ± 61,770.6 reads per sample that were assigned to 7.8 ± 3.5 different fungal genera (Figures S1 and S2). In the Mesa site, the animal-associated dataset has an average of 340.1 ± 923.8 reads per sample that were assigned to 4.6 ± 3.4 different genera (Figures S1 and S2). Beta and alpha diversity comparisons had the same results found in the analyses of the whole dataset. No significant effects of *Coccidioides* presence (*p* = 0.184), host genus (*p* = 0.442) and host family (*p* = 0.488) in alpha diversity. Host genus (*p* = 0.001) and host family (*p* = 0.001) have a significant effect on beta diversity.

## 4. Discussion

In this study, we describe the prevalence of *Coccidioides* in mammal lung samples from sites in Tucson and Mesa, Arizona, USA. We also describe the mycobiome in these samples and compare fungal community diversity between *Coccidioides*-positive and -negative at order and genus levels. We found that small mammals in endemic areas carry a diverse community of fungi in their lungs, but no significant differences were found between *Coccidioides*-positive and -negative samples, suggesting that *Coccidioides* does not significantly alter the lung mycobiome in these hosts.

In both sites, most OTU were assigned to the order Onygenales. Tucson site samples exhibited lower *Coccidioides* prevalence, with only certain host genera (i.e., *Chaetodipus*, *Onychomys*, *Ammospermophilus* and *Sylvilagus*) testing positive for *Coccidioides*. In contrast, *Coccidioides* was highly prevalent in the lungs of mammals from the Mesa site, where all *Peromyscus* tested positive for *Coccidioides*. Given that all Mesa site samples were *Coccidioides* -positive, and considering the limited sequencing depth and overall low read counts, no statistical analyses were performed using this dataset. We suspect these limitations may be due to issues during the DNA extraction or the library preparation.

In terms of mycobiome diversity, the Mesa site revealed a lung mycobiome dominated by *Coccidioides* (78.7% of the reads), with other common genera including *Epicoccum*, *Toxicocladosporium*, and *Alternaria*. The Tucson site exhibited a more even distribution of fungal genera, with taxa such as *Ajellomyces*, *Chaetomium*, and *Rhizopus* predominating in the lungs. These findings align with what was found in soil samples from these sites. In Tucson, 10.4% of the burrow-associated soils screened in this study were positive (Table S1). In Mesa, 43% of soil samples were positive when collected near rodent burrows [46].

Host taxonomy was associated with differences in beta diversity, suggesting that host physiology and behavior might shape the lung mycobiome of mammals obtained in the same location [66]. However, it is important to note that host genera were identified based on morphological characteristics which can lead to misidentification [3,67]. There were no significant differences in alpha and beta diversity between *Coccidioides*-positive and -negative samples. This result aligns with previous research that showed no differences in the mycobiome of *Coccidioides*-positive and -negative lungs from museum specimens [2]. The absence of differences in the lung mycobiome of *Coccidioides*-positive and -negative samples, suggests that *Coccidioides* is behaving as a commensal, as pathogens are expected to be associated with shifts in the microbiome [5,6,7]. Therefore, our fungal diversity results are consistent with the endozoan hypothesis which states that the fungus will persist in small-mammal hosts from endemic areas without causing disease [40].

Studying the mycobiome in the lungs is challenging and given the importance of detecting *Coccidioides* for the objective of this study, we used two methods to ensure detection: CocciDx and an Illumina-based ITS2 amplicon sequencing. Although both methods successfully detected *Coccidioides* presence, CocciDx detected *Coccidioides* in 19 samples, while the ITS2 amplicon sequencing detected *Coccidioides* in six samples. ITS2 amplicon sequencing is a powerful tool to characterize fungal diversity, but it can underestimate or overestimate certain taxa [50]. In contrast, CocciDx is a qPCR-based assay that targets a *Coccidioides*-specific sequence [48,49]. This result highlights the limitations of using a single detection method when looking for a specific genus or species.

The dynamicity of the lung environment leads to the presence of organisms that are not part of the established mycobiome, but only transiently present in the lung [10,11]. However, in this study, the presence of potentially transient genera did not seem to have an impact on our dataset and analyses. Once we excluded the fungal genera not previously known to be associated with vertebrate animals (e.g., associated with plant, feces, soil, etc.), we still retained most of the OTUs. In the Mesa site samples, animal-associated fungal genera accounted for 89.2% of the reads and 88.3% of the reads in Tucson. Additionally, most of the genera within the ten most prevalent were retained, especially those with higher number of reads. The alpha and beta diversity comparisons of the animal-associated dataset had the same outcome as the whole dataset analysis. Host taxonomy has a significant effect on mycobiome beta diversity, but *Coccidioides* presence does not. We conclude that fungal genera that were transiently present in the lungs did not affect the outcome of our study. We hypothesize this is because the noise caused by these fungi is similar across all individuals obtained in the same site.

We acknowledge that conclusions from our study are limited by its cross-sectional nature, small sample size and the lack of information on the specimens from which the lung samples were obtained. Studies have shown that different factors can shape the lung microbiome including age and disease progression [10,68,69,70]. Although we have not observed an effect of *Coccidioides* presence in the lung mycobiome, *Coccidioides* could be affecting the diversity of viruses and bacteria which our study did not characterize. Moreover, we do not have information on the timing of infection by *Coccidioides* or by any other pathogens for these individuals. The timing of *Coccidioides* infection and presence of other pathogens could play a role in the lack of mycobiome differences between *Coccidioides*-positive and -negative samples. Also, time since death and freeze/thawing cycles could have caused changes in the mycobiome profiles [71,72].

Future studies could benefit from obtaining samples for which life history information is available. This could be achieved by obtaining samples from zoos, animal diversity management programs, and farms. Additionally, a better understanding of the behavior of *Coccidioides* in the lungs and its effects on the lung mycobiome could come from laboratory studies comparing the mycobiome of mice from the same background under controlled experimental conditions.

## Figures and Tables

**Table 1:** *Coccidioides* positivity in lung samples comparing qPCR (CocciDx) C_T_ values and amplicon sequencing results.

Site	Host Species	ID	C_T_ Value (CocciDx)	Amplicon Count ^1^
Tucson	*Chaetodipus*	PM_4	31	56 (0.04%)
		PM_5	32	111 (0.18%)
	*Sylvilagus*	CT_6	38.7	
	*Onychomys*	GH_M	39.6	
	*Ammospermophilus*	A_GS	39.8	
Mesa	*Peromyscus*	17L	38.9	
		24L	34.5	
		29L	38.5	
		31L	28.6	
		33L	36.3	9 (11.4%)
		34L	31.5	431(60.4%)
		35L	31.5	3606 (95.9%)
		36L	33.7	
		37L	34.1	
		38L	36.4	193 (89.8%)
		39L	31.7	
		40L	34.1	
		41L	33.3	
		43L	33.8	

^1^ Total amplicon reads (Relative abundance).

## Data Availability

The original contributions presented in this study are included in the article/Supplementary Materials. Further inquiries can be directed to the corresponding author.

## Supplementary Materials

The following supporting information can be downloaded at: https://www.mdpi.com/article/10.3390/pathogens14080807/s1, Figure S1: Animal-associated fungal genera in lung mycobiome plotted for each site when samples are positive or negative for Coccidioides; Figure S2: Animal-associated fungal genera in lung mycobiome plotted for each host genus when they are negative or positive for *Coccidioides*. Supplement Table S1. Coccidioides positivity in soil samples collected near rodent burrows from Tucson, AZ Spreadsheet S1. FunGuild amended database, and ITS2 amplicon sequencing reads assigned to fungal genus.

## References

[1] Melo-Neto M.F., Maranhão F.C.A., Guimarães P.S., Silva D.M.W. (2022). Mycobiota recovered from the trachea and lungs of pigeons (Columba livia) captured in a grain mill. Res. Soc. Dev..

[2] Salazar-Hamm P.S., Gadek C.R., Mann M.A., Steinberg M., Montoya K.N., Behnia M., Gyllenhaal E.F., Brady S.S., Takano O.M., Williamson J.L. (2025). Phylogenetic and ecological drivers of the avian lung mycobiome and its potentially pathogenic component. Commun. Biol..

[3] Salazar-Hamm P.S., Montoya K.N., Montoya L., Cook K., Liphardt S., Taylor J.W., Cook J.A., Natvig D.O. (2022). Breathing can be dangerous: Opportunistic fungal pathogens and the diverse community of the small mammal lung mycobiome. Front. Fungal Biol..

[4] Babb-Biernacki S.J., Esselstyn J.A., Doyle V.P. (2022). Predicting Species Boundaries and Assessing Undescribed Diversity in Pneumocystis, an Obligate Lung Symbiont. J. Fungi.

[5] Allender M.C., Baker S., Britton M., Kent A.D. (2018). Snake fungal disease alters skin bacterial and fungal diversity in an endangered rattlesnake. Sci. Rep..

[6] Jani A.J., Briggs C.J. (2014). The pathogen Batrachochytrium dendrobatidis disturbs the frog skin microbiome during a natural epidemic and experimental infection. Proc. Natl. Acad. Sci. USA.

[7] Lloyd M.M., Pespeni M.H. (2018). Microbiome shifts with onset and progression of Sea Star Wasting Disease revealed through time course sampling. Sci. Rep..

[8] Richardson H., Dicker A.J., Barclay H., Chalmers J.D. (2019). The microbiome in bronchiectasis. Eur. Respir. Rev..

[9] Richardson M., Bowyer P., Sabino R. (2019). The human lung and Aspergillus: You are what you breathe in?. Med. Mycol..

[10] Li R., Li J., Zhou X. (2024). Lung microbiome: New insights into the pathogenesis of respiratory diseases. Signal Transduct. Target. Ther..

[11] Natalini J.G., Singh S., Segal L.N. (2023). The dynamic lung microbiome in health and disease. Nat. Rev. Microbiol..

[12] Hamm P.S., Taylor J.W., Cook J.A., Natvig D.O. (2020). Decades-old studies of fungi associated with mammalian lungs and modern DNA sequencing approaches help define the nature of the lung mycobiome. PLoS Pathog..

[13] Penders J., Thijs C., Vink C., Stelma F.F., Snijders B., Kummeling I., van den Brandt P.A., Stobberingh E.E. (2006). Factors Influencing the Composition of the Intestinal Microbiota in Early Infancy. Pediatrics.

[14] Mitchell C.M., Mazzoni C., Hogstrom L., Bryant A., Bergerat A., Cher A., Pochan S., Herman P., Carrigan M., Sharp K. (2020). Delivery Mode Affects Stability of Early Infant Gut Microbiota. Cell Rep. Med..

[15] Reyman M., van Houten M.A., van Baarle D., Bosch A.A.T.M., Man W.H., Chu M.L.J.N., Arp K., Watson R.L., Sanders E.A.M., Fuentes S. (2019). Impact of delivery mode-associated gut microbiota dynamics on health in the first year of life. Nat. Commun..

[16] Badal V.D., Vaccariello E.D., Murray E.R., Yu K.E., Knight R., Jeste D.V., Nguyen T.T. (2020). The Gut Microbiome, Aging, and Longevity: A Systematic Review. Nutrients.

[17] Bartley J.M., Zhou X., Kuchel G.A., Weinstock G.M., Haynes L. (2017). Impact of Age, Caloric Restriction, and Influenza Infection on Mouse Gut Microbiome: An Exploratory Study of the Role of Age-Related Microbiome Changes on Influenza Responses. Front. Immunol..

[18] Emmons C.W., Ashburn L.L. (1942). The Isolation of Haplosporangium parvum n. sp. and *Coccidioides immitis* from Wild Rodents. Their Relationship to Coccidioidomycosis. Public Health Rep. (1896–1970).

[19] Emmons C.W. (1943). Coccidioidomycosis in Wild Rodents. A Method of Determining the Extent of Endemic Areas. Public Health Rep. (1896–1970).

[20] Bakerspigel A. (1956). Haplosporangium in Saskatchewan Rodents. Mycologia.

[21] Jellison W.L. (1950). Haplomycosis in Montana Rabbits, Rodents, and Carnivores. Public Health Rep. (1896–1970).

[22] Delhaes L., Monchy S., Fréalle E., Hubans C., Salleron J., Leroy S., Prevotat A., Wallet F., Wallaert B., Dei-Cas E. (2012). The Airway Microbiota in Cystic Fibrosis: A Complex Fungal and Bacterial Community—Implications for Therapeutic Management. PLoS ONE.

[23] Kollath D.R., Dolby G.A., Webster T.H., Barker B.M. (2023). Characterizing fungal communities on Mojave desert tortoises (*Gopherus agassizii*) in Arizona to uncover potential pathogens. J. Arid Environ..

[24] Zhang H., Zhang K., Ma H., Deng J., Fang C., Zhao H., An X., Zhang J., Wang Q., Jiang W. (2025). Seasonality Has Greater Influence on Amphibian Cutaneous Mycobiome than Host Species. J. Fungi.

[25] Kearns P.J., Fischer S., Fernández-Beaskoetxea S., Gabor C.R., Bosch J., Bowen J.L., Tlusty M.F., Woodhams D.C. (2017). Fight Fungi with Fungi: Antifungal Properties of the Amphibian Mycobiome. Front. Microbiol..

[26] Kearns P.J., Winter A.S., Woodhams D.C., Northup D.E. (2023). The Mycobiome of Bats in the American Southwest Is Structured by Geography, Bat Species, and Behavior. Microb. Ecol..

[27] Weinstein S.B., Stephens W.Z., Greenhalgh R., Round J.L., Dearing M.D. (2022). Wild herbivorous mammals (genus Neotoma) host a diverse but transient assemblage of fungi. Symbiosis.

[28] Vargas-Gastélum L., Romer Alexander S., Ghotbi M., Dallas Jason W., Alexander N.R., Moe Kylie C., McPhail Kerry L., Neuhaus George F., Shadmani L., Spatafora Joseph W. (2024). Herptile gut microbiomes: A natural system to study multi-kingdom interactions between filamentous fungi and bacteria. mSphere.

[29] Hathaway J.J.M., Salazar-Hamm P.S., Caimi N.A., Natvig D.O., Buecher D.C., Northup D.E. (2024). Comparison of Fungal and Bacterial Microbiomes of Bats and Their Cave Roosting Environments at El Malpais National Monument, New Mexico, USA. Geomicrobiol. J..

[30] Zapanta K., Kavanagh M., Keller K., Nguyen L., Rosenkrantz W., Krumbeck J.A. (2025). The cutaneous microbiota and Nannizziomycosis in bearded dragons (*Pogona vitticeps*): Associations between infectious *Nannizziopsis* species and common bacterial pathogens. Vet. Dermatol..

[31] Insuk C., Cheeptham N., Lausen C., Xu J. (2024). DNA metabarcoding analyses reveal fine-scale microbiome structures on Western Canadian bat wings. Microbiol. Spectr..

[32] Fisher M.C., Koenig G.L., White T.J., Taylor J.W. (2002). Molecular and phenotypic description of *Coccidioides posadasii* sp. nov., previously recognized as the non-California population of *Coccidioides immitis*. Mycologia.

[33] de Perio M., Niemeier R.T., Burr G. (2015). *Coccidioides* Exposure and Coccidioidomycosis among Prison Employees, California, United States. Emerg. Infect. Dis. J..

[34] Wack E.E., Ampel N.M., Sunenshine R.H., Galgiani J.N. (2015). The Return of Delayed-Type Hypersensitivity Skin Testing for Coccidioidomycosis. Clin. Infect. Dis..

[35] Drips W., Smith C.E. (1964). Epidemiology of Coccidioidomycosis: A Contemporary Military Experience. JAMA.

[36] Smith C.E., Saito M.T., Simons S.A. (1956). Pattern of 39,500 serologic tests in coccidioidomycosis. J. Am. Med. Assoc..

[37] CDC C.f.D.C.a.P. Valley Fever (Coccidioidomycosis). https://www.cdc.gov/valley-fever/php/statistics/index.html.

[38] Grizzle A.J., Wilson L., Nix D.E., Galgiani J.N. (2021). Clinical and Economic Burden of Valley Fever in Arizona: An Incidence-Based Cost-of-Illness Analysis. Open Forum Infect. Dis..

[39] Barker B.M., Seyedmousavi S., de Hoog G.S., Guillot J., Verweij P.E. (2018). Coccidioidomycosis in Animals. Emerging and Epizootic Fungal Infections in Animals.

[40] Taylor J.W., Barker B.M. (2019). The endozoan, small-mammal reservoir hypothesis and the life cycle of *Coccidioides* species. Med. Mycol..

[41] Andreote F.D., Gumiere T., Durrer A. (2014). Exploring interactions of plant microbiomes. Sci. Agrícola.

[42] Elconin A.F., Egeberg R.O., Egeberg M.C. (1964). Significance of soil salinity on the ecology of *Coccidioides immitis*. J. Bacteriol..

[43] Lacy G.H., Swatek F.E. (1974). Soil Ecology of *Coccidioides immitis* at Amerindian Middens in California. Appl. Microbiol..

[44] Chow N.A., Kangiser D., Gade L., McCotter Orion Z., Hurst S., Salamone A., Wohrle R., Clifford W., Kim S., Salah Z. (2021). Factors Influencing Distribution of *Coccidioides immitis* in Soil, Washington State, 2016. mSphere.

[45] Kollath D.R., Teixeira M.M., Funke A., Miller K.J., Barker B.M. (2020). Investigating the Role of Animal Burrows on the Ecology and Distribution of *Coccidioides* spp. in Arizona Soils. Mycopathologia.

[46] Ramsey M., Barker Bridget M. (2025). Investigating Prevalence of Coccidioides Over 3 Years (2022–2025) at a Single Site in Mesa, Arizona, United States.

[47] Lauer A., Baal J.D.H., Baal J.C.H., Verma M., Chen J.M. (2012). Detection of *Coccidioides immitis* in Kern County, California, by multiplex PCR. Mycologia.

[48] Bowers J.R., Parise K.L., Kelley E.J., Lemmer D., Schupp J.M., Driebe E.M., Engelthaler D.M., Keim P., Barker B.M. (2019). Direct detection of *Coccidioides* from Arizona soils using CocciENV, a highly sensitive and specific real-time PCR assay. Med. Mycol..

[49] Litvintseva A.P., Marsden-Haug N., Hurst S., Hill H., Gade L., Driebe E.M., Ralston C., Roe C., Barker B.M., Goldoft M. (2015). Valley Fever: Finding New Places for an Old Disease: *Coccidioides immitis* Found in Washington State Soil Associated with Recent Human Infection. Clin. Infect. Dis..

[50] Taylor D.L., Walters William A., Lennon Niall J., Bochicchio J., Krohn A., Caporaso J.G., Pennanen T. (2016). Accurate Estimation of Fungal Diversity and Abundance through Improved Lineage-Specific Primers Optimized for Illumina Amplicon Sequencing. Appl. Environ. Microbiol..

[51] Colman R.E., Schupp J.M., Hicks N.D., Smith D.E., Buchhagen J.L., Valafar F., Crudu V., Romancenco E., Noroc E., Jackson L. (2015). Detection of Low-Level Mixed-Population Drug Resistance in Mycobacterium tuberculosis Using High Fidelity Amplicon Sequencing. PLoS ONE.

[52] Martin T., Lu S.-W., van Tilbeurgh H., Ripoll D.R., Dixelius C., Turgeon B.G., Debuchy R. (2010). Tracing the Origin of the Fungal α1 Domain Places Its Ancestor in the HMG-Box Superfamily: Implication for Fungal Mating-Type Evolution. PLoS ONE.

[53] Bolyen E., Rideout J.R., Dillon M.R., Bokulich N.A., Abnet C.C., Al-Ghalith G.A., Alexander H., Alm E.J., Arumugam M., Asnicar F. (2019). Reproducible, interactive, scalable and extensible microbiome data science using QIIME 2. Nat. Biotechnol..

[54] Callahan B.J., McMurdie P.J., Rosen M.J., Han A.W., Johnson A.J.A., Holmes S.P. (2016). DADA2: High-resolution sample inference from Illumina amplicon data. Nat. Methods.

[55] Rognes T., Flouri T., Nichols B., Quince C., Mahé F. (2016). VSEARCH: A versatile open source tool for metagenomics. PeerJ.

[56] Bengtsson-Palme J., Ryberg M., Hartmann M., Branco S., Wang Z., Godhe A., De Wit P., Sánchez-García M., Ebersberger I., de Sousa F. (2013). Improved software detection and extraction of ITS1 and ITS2 from ribosomal ITS sequences of fungi and other eukaryotes for analysis of environmental sequencing data. Methods Ecol. Evol..

[57] Van Nguyen H., Lavenier D. (2009). PLAST: Parallel local alignment search tool for database comparison. BMC Bioinform..

[58] Hibbett D., Abarenkov K., Kõljalg U., Öpik M., Chai B., Cole J., Wang Q., Crous P., Robert V., Helgason T. (2016). Sequence-based classification and identification of Fungi. Mycologia.

[59] Nguyen N.H., Song Z., Bates S.T., Branco S., Tedersoo L., Menke J., Schilling J.S., Kennedy P.G. (2016). FUNGuild: An open annotation tool for parsing fungal community datasets by ecological guild. Fungal Ecol..

[60] Lahti L., Shetty S. (2017). Microbiome R Package, Bioconductor. https://github.com/microbiome/microbiome.

[61] McMurdie P.J., Holmes S. (2013). phyloseq: An R Package for Reproducible Interactive Analysis and Graphics of Microbiome Census Data. PLoS ONE.

[62] Wickham H. (2016). ggplot2: Elegant Graphics for Data Analysis.

[63] Oksanen J., Blanchet F.G., Friendly M., Kindt R., Legendre P., Minchin P.R., O’Hara R.B., Solymos P., McGlinn D., Szoecs E. (2019). Vegan: Community Ecology Package, 2.5-6. https://cran.r-project.org/web/packages/vegan/index.html.

[64] Wickham H., Averick M., Bryan J., Chang W., McGowan L.D.A., François R., Grolemund G., Hayes A., Henry L., Hester J. (2019). Welcome to the tidyverse. J. Open Source Softw..

[65] Wickham H., François R., Henry L., Müller K., Vaughan D. (2023). dplyr: A Grammar of Data Manipulation. https://github.com/tidyverse/dplyr.

[66] Araujo G., Montoya J.M., Thomas T., Webster N.S., Lurgi M. (2025). A mechanistic framework for complex microbe-host symbioses. Trends Microbiol..

[67] Müller L., Gonçalves G.L., Cordeiro-Estrela P., Marinho J.R., Althoff S.L., Testoni A.F., González E.M., Freitas T.R.O. (2013). DNA Barcoding of Sigmodontine Rodents: Identifying Wildlife Reservoirs of Zoonoses. PLoS ONE.

[68] Madan J.C., Koestler D.C., Stanton B.A., Davidson L., Moulton L.A., Housman M.L., Moore J.H., Guill M.F., Morrison H.G., Sogin M.L. (2012). Serial Analysis of the Gut and Respiratory Microbiome in Cystic Fibrosis in Infancy: Interaction between Intestinal and Respiratory Tracts and Impact of Nutritional Exposures. mBio.

[69] Coburn B., Wang P.W., Diaz Caballero J., Clark S.T., Brahma V., Donaldson S., Zhang Y., Surendra A., Gong Y., Elizabeth Tullis D. (2015). Lung microbiota across age and disease stage in cystic fibrosis. Sci. Rep..

[70] Chen R., Wang L., Koch T., Curtis V., Yin-DeClue H., Handley S.A., Shan L., Holtzman M.J., Castro M., Wang L. (2020). Sex effects in the association between airway microbiome and asthma. Ann. Allergy Asthma Immunol..

[71] Moitas B., Caldas I.M., Sampaio-Maia B. (2024). Microbiology and postmortem interval: A systematic review. Forensic Sci. Med. Pathol..

[72] Pechal J.L., Schmidt C.J., Jordan H.R., Benbow M.E. (2017). Frozen: Thawing and Its Effect on the Postmortem Microbiome in Two Pediatric Cases. J. Forensic Sci..

