# Supplementary material for: Dancing with the Dust Devil: Examining the Lung Mycobiome of Sonoran Desert Wild Mammals and the Effect of Coccidioides Presence
Source: Pathogens. 2025 Aug 14;14(8):807. doi: 10.3390/pathogens14080807 (PMC12388877; doi:10.3390/pathogens14080807)
Supplement: Supplementary file 1 [file pathogens-14-00807-s001.zip › Figure_legends_supplemental.pdf]

Figure S1. Animal-associated fungal genera in lung mycobiome plotted for each site when samples are positive or negative for *Coccidioides*. The most prevalent genera in the Tucson site (n=26) were *Ajellomyces* (39.1%), *Chaetomium* (12.2%), *Rhizopus* (8.0%), *Naganishia* (7.5%), *Candida* (5.7%), *Alternaria* (5.2%), *Penicillium* (5.1%), *Kluyveromyces* (4.4%), *Pneumocystis* (3.8%), and *Filobasidium* (3.2%, Figure 8). The most prevalent genera in the Mesa site (n=14) were *Coccidioides* (89.0%), *Epicoccum* (6.2%), *Alternaria* (2.2%), *Trematosphaeria* (0.7%), *Curvularia* (0.3%), *Didymella* (0.3%), *Circinella* (0.3%), *Mortierella* (0.2%), *Aureobasidium* (0.1%), and *Trichocladium* (0.1%).

Figure S2. Animal-associated fungal genera in lung mycobiome plotted for each host genus when they are negative or positive for *Coccidioides*. In ***Dipodomys*** (n=1) the most abundant species were *Rhizopus* (81.9%), *Chaetomium* (14.5%), *Penicillium* (1.6%), *Humicola* (1.0%), *Aspergillus* (0.9%); in ***Onychomys*** (n=1), *Chaetomium* (85.6%), *Rhizopus* (11.7%), *Filobasidium* (1.7%), *Penicillium* (1.0%); in ***Ammospermophilus*** (n=1), Ajellomyces (100%); in ***Xerospermophilus*** (n=1), *Candida* (78.3%), *Pneumocystis* (14.3%), *Naganishia* (4.6%), *Aureobasidium* (2.4%), *Chaetomium* (0.4%); in ***Chaetodipus*** (n=12), *Ajellomyces* (74.1%), *Kluyveromyces* (6.3%), *Pneumocystis* (5.8%), *Alternaria* (4.5%), *Penicillium* (2.8%); in ***Sylvilagus*** (n=6), *Naganishia* (28.9%), *Rhizopus* (15.5%), *Chaetomium* (11.9%), *Penicillium* (11.9%), *Filobasidium* (11.9%); in ***Lepus*** (n=4), *Alternaria* (33.1%), *Candida* (22.9%), *Rhizopus* (18.6%), *Chaetomium* (9.1%), *Penicillium* (8.8%). In ***Peromyscus*** (n=14; Mesa site), the most abundant genera are *Coccidioides* (89.0%), *Epicoccum* (6.2%), *Alternaria* (2.2%), *Trematosphaeria* (0.7%), *Curvularia* (0.3%), *Didymella* (0.3%), *Circinella* (0.3%), *Mortierella* (0.2%), *Aureobasidium* (0.1%), and *Trichocladium* (0.1%).
