# Supplementary material for: Dancing with the Dust Devil: Examining the Lung Mycobiome of Sonoran Desert Wild Mammals and the Effect of Coccidioides Presence
Source: Pathogens. 2025 Aug 14;14(8):807. doi: 10.3390/pathogens14080807 (PMC12388877; doi:10.3390/pathogens14080807)
Supplement: Supplementary file 1 [file pathogens-14-00807-s001.zip › Table_S1_CT_soils.pdf]

Table S1. *Coccidioides* positivity in soil samples collected near rodent burrows from Tucson, AZ.

Soil sample ID[1]	C_T Value (CocciDx)[2]
**B1-2i**	39.34
**B1-3ii**	39.22
**B2-1i**	39.78
**B2-3**	39.32
**B2-4**	37.8

[1]Positive samples only [2]Measured by CocciDx qPCR assay (Bowers et al. 2019).
